# MethBank 4.0: an updated database of DNA methylation across a variety of species

**DOI:** 10.1093/nar/gkac969

**Published:** 2022-11-01

**Authors:** Mochen Zhang, Wenting Zong, Dong Zou, Guoliang Wang, Wei Zhao, Fei Yang, Song Wu, Xinran Zhang, Xutong Guo, Yingke Ma, Zhuang Xiong, Zhang Zhang, Yiming Bao, Rujiao Li

**Affiliations:** National Genomics Data Center & CAS Key Laboratory of Genome Sciences and Information, Beijing Institute of Genomics, Chinese Academy of Sciences and China National Center for Bioinformation, Beijing 100101, China; China National Center for Bioinformation, Beijing 100101, China; University of Chinese Academy of Sciences, Beijing 100049, China; National Genomics Data Center & CAS Key Laboratory of Genome Sciences and Information, Beijing Institute of Genomics, Chinese Academy of Sciences and China National Center for Bioinformation, Beijing 100101, China; China National Center for Bioinformation, Beijing 100101, China; University of Chinese Academy of Sciences, Beijing 100049, China; National Genomics Data Center & CAS Key Laboratory of Genome Sciences and Information, Beijing Institute of Genomics, Chinese Academy of Sciences and China National Center for Bioinformation, Beijing 100101, China; China National Center for Bioinformation, Beijing 100101, China; National Genomics Data Center & CAS Key Laboratory of Genome Sciences and Information, Beijing Institute of Genomics, Chinese Academy of Sciences and China National Center for Bioinformation, Beijing 100101, China; China National Center for Bioinformation, Beijing 100101, China; University of Chinese Academy of Sciences, Beijing 100049, China; National Genomics Data Center & CAS Key Laboratory of Genome Sciences and Information, Beijing Institute of Genomics, Chinese Academy of Sciences and China National Center for Bioinformation, Beijing 100101, China; China National Center for Bioinformation, Beijing 100101, China; University of Chinese Academy of Sciences, Beijing 100049, China; National Genomics Data Center & CAS Key Laboratory of Genome Sciences and Information, Beijing Institute of Genomics, Chinese Academy of Sciences and China National Center for Bioinformation, Beijing 100101, China; China National Center for Bioinformation, Beijing 100101, China; National Genomics Data Center & CAS Key Laboratory of Genome Sciences and Information, Beijing Institute of Genomics, Chinese Academy of Sciences and China National Center for Bioinformation, Beijing 100101, China; China National Center for Bioinformation, Beijing 100101, China; University of Chinese Academy of Sciences, Beijing 100049, China; National Genomics Data Center & CAS Key Laboratory of Genome Sciences and Information, Beijing Institute of Genomics, Chinese Academy of Sciences and China National Center for Bioinformation, Beijing 100101, China; China National Center for Bioinformation, Beijing 100101, China; University of Chinese Academy of Sciences, Beijing 100049, China; National Genomics Data Center & CAS Key Laboratory of Genome Sciences and Information, Beijing Institute of Genomics, Chinese Academy of Sciences and China National Center for Bioinformation, Beijing 100101, China; China National Center for Bioinformation, Beijing 100101, China; University of Chinese Academy of Sciences, Beijing 100049, China; National Genomics Data Center & CAS Key Laboratory of Genome Sciences and Information, Beijing Institute of Genomics, Chinese Academy of Sciences and China National Center for Bioinformation, Beijing 100101, China; China National Center for Bioinformation, Beijing 100101, China; National Genomics Data Center & CAS Key Laboratory of Genome Sciences and Information, Beijing Institute of Genomics, Chinese Academy of Sciences and China National Center for Bioinformation, Beijing 100101, China; China National Center for Bioinformation, Beijing 100101, China; University of Chinese Academy of Sciences, Beijing 100049, China; National Genomics Data Center & CAS Key Laboratory of Genome Sciences and Information, Beijing Institute of Genomics, Chinese Academy of Sciences and China National Center for Bioinformation, Beijing 100101, China; China National Center for Bioinformation, Beijing 100101, China; University of Chinese Academy of Sciences, Beijing 100049, China; National Genomics Data Center & CAS Key Laboratory of Genome Sciences and Information, Beijing Institute of Genomics, Chinese Academy of Sciences and China National Center for Bioinformation, Beijing 100101, China; China National Center for Bioinformation, Beijing 100101, China; University of Chinese Academy of Sciences, Beijing 100049, China; National Genomics Data Center & CAS Key Laboratory of Genome Sciences and Information, Beijing Institute of Genomics, Chinese Academy of Sciences and China National Center for Bioinformation, Beijing 100101, China; China National Center for Bioinformation, Beijing 100101, China; University of Chinese Academy of Sciences, Beijing 100049, China

## Abstract

DNA methylation, as the most intensively studied epigenetic mark, regulates gene expression in numerous biological processes including development, aging, and disease. With the rapid accumulation of whole-genome bisulfite sequencing data, integrating, archiving, analyzing, and visualizing those data becomes critical. Since its first publication in 2015, MethBank has been continuously updated to include more DNA methylomes across more diverse species. Here, we present MethBank 4.0 (https://ngdc.cncb.ac.cn/methbank/), which reports an increase of 309% in data volume, with 1449 single-base resolution methylomes of 23 species, covering 236 tissues/cell lines and 15 biological contexts. Value-added information, such as more rigorous quality evaluation, more standardized metadata, and comprehensive downstream annotations have been integrated in the new version. Moreover, expert-curated knowledge modules of featured differentially methylated genes associated with biological contexts and methylation analysis tools have been incorporated as new components of MethBank. In addition, MethBank 4.0 is equipped with a series of new web interfaces to browse, search, and visualize DNA methylation profiles and related information. With all these improvements, we believe the updated MethBank 4.0 will serve as a fundamental resource to provide a wide range of data services for the global research community.

## INTRODUCTION

As a stable epigenetic modification, DNA methylation plays a crucial role in gene regulation and genome stability in various biological processes (1). Aberrant DNA methylation patterns in cancers and other diseases are promising diagnostic, prognostic, and therapeutic biomarkers (2). In addition, DNA methylation patterns of parental chromatins show dynamic erasure and re-establishment during developments in posteriors animals and plants (3–5), guiding the screening of production traits in domestic livestock and cash crops. With the continuous increase in the studies of DNA methylation at single-base resolution, a wealth of whole-genome bisulfite sequencing (WGBS) data and methylation research results in multiple biological contexts have been published. At the same time, a large number of analysis tools have also been developed. Methods to better integrate and standardize these public data, extract and organize these precious research results, and collate and summarize a variety of data analysis tools, are of great importance for further studies and applications of DNA methylation. Therefore, it puts higher demands on the construction of the corresponding database. Despite several efforts of the extant WGBS methylation databases, there are still some deficiencies that need to be addressed, such as restricted range of samples, limited frequency of updates and maintenance, and lack of knowledge integration based on sequencing data. For instance, NGSmethDB (6) stores <200 samples from seven species, MethDB (7) has not been updated since 22 September 2009, while ChIP-Atlas (8) collects 51 074 methylomes for the six representative model organisms without metadata integration. In light of the above, there is an urgent need for a comprehensive database integrating data resources, collecting knowledge, and developing tools of DNA methylation to help researchers reveal the regulatory mechanisms of DNA methylation.

With the previous versions released in 2014 (9) and 2018 (10) respectively, MethBank (https://ngdc.cncb.ac.cn/methbank/) has undergone continuous maintenance and regular updates over the past eight years to integrate, archive, and visualize public whole-genome DNA methylation data, as well as to curate, normalize, and correlate characteristic methylation features in various biological contexts. As a valuable data resource for DNA methylation, MethBank 3.0 promotes a deeper understanding of the regulatory mechanisms of DNA methylation in animals (11,12) and plants (13,14). In order to accommodate more data and knowledge to better support methylation-related research, here we introduce an updated implementation of MethBank 4.0 and describe its significant improvements. Specifically, MethBank 4.0 dramatically increases the amount of existing data and enhances the functions for data annotation, greatly expanding the resources of potential epigenetic features in 15 biological contexts including diseases, aging, developments, genotypes, environments, and tissue comparations. Moreover, to provide users with systematically summarized and standardized information on published DNA methylation research results, MethBank 4.0 not only provides a manually curated collection of featured differentially methylated genes (DMGs) across multiple biological contexts, but also collects a wealth of information on publicly released methylation analysis tools. Furthermore, a stand-alone tool named DMR Toolkit is equipped to support the identification, annotation, and visualization of differentially methylated regions (DMRs), thus helping users to find features of DNA methylation in a particular biological context. With the addition of large-scale whole-genome single-base resolution methylomes, methylation-related knowledge and the DMR Toolkit, web interfaces of MethBank are accordingly updated to facilitate users to easily access and retrieve information of interest.

## NEW FEATURES AND UPDATES

### Data module

Since the release of version 3.0, MethBank has greatly increased the breadth and depth of data content while maintaining quality and reliability (Table 1). This is reflected in three aspects: (i) MethBank 4.0 enormously increases the quantity of whole-genome single-base resolution methylomes that are obtained using a standardized analysis workflow; (ii) MethBank 4.0 adopts a structured metadata curation model to integrate the descriptive information about both projects and samples; (iii) MethBank 4.0 not only systematically identifies and annotates DMRs, but also simultaneously integrates standard downstream analysis for gene methylation profiles and methylated CpG islands (mCpGIs) released in the previous version.

**Table 1.** Significant improvements of MethBank 4.0

**Modules**	**Item**	**MethBank 4.0 (updated)**	**MethBank 3.0 (2018)**
**Data**	Species	23	7
	Projects	199	20
	Metadata curation of projects	1 section/18 items	NA
	Samples	1449	354
	Metadata curation of samples	6 sections/45 items	NA
	Tissues/cell lines	236	42
	Biological contexts	15	NA
	Gene methylation profiles	67 866 168	4 594 320
	Methylated CpG islands	33 757 558	693 825
	Genes related to mCpGIs^a^	7 612 395	137 018
	Differentially methylated regions	59 750 225	NA
	Genes related to DMRs^b^	258 628	NA
**Knowledge**			
Featured DMGs	Species	2	NA
	Tissues/cell lines	77	NA
	Biological contexts	11	NA
	Diseases	128	NA
	Publications	228	NA
Tool Collection	Tools	501	NA
	Items	25	NA
	Publications	480	NA
**Tools**	Age predictor	Yes	Yes
	IDMP	Yes	Yes
	DMR toolkit	Yes	NA

^a^Methylated CpG islands.

^b^Differentially methylated regions.

MethBank 4.0 incorporates more projects and samples that cover a wider range of species and biological contexts. The current version houses whole-genome single-base resolution methylomes in CG, CHG, and CHH (H = A, T or C) sequence contexts from 1449 higher quality WGBS data in 199 projects from 23 species, covering 236 tissues/cell lines and 15 biological contexts, representing a dramatic increase in data content by 309% compared with the version 3.0. Specifically, MethBank 4.0 extracts 120 high-quality datasets from all collected projects to facilitate users to easily screen high-quality sample collections. In contrast to the previous release that included only 2 animal species and 5 plant species, MethBank 4.0 additionally incorporates 16 animal species and 7 plant species, which enables easy access to a comprehensive range of DNA methylation profiles from multiple species. The newly added species include *Homo sapiens*, four non-human primates (*Pan troglodytes*, *Gorilla gorilla*, *Macaca mulatta* and *Macaca fascicularis*), two model organisms (*Rattus norvegicus* and *Xenopus laevis*), five domesticated animals (*Gallus gallus*, *Bos taurus*, *Sus scrofa*, *Salmo salar* and *Ovis aries*), one companion animal (*Canis lupus familiaris*), one endangered mammal (*Ailuropoda melanoleuca*) and two cultivated plants (*Zea mays* and *Brassica napus*). Notably, in order to facilitate users to compare data from other sources, MethBank 4.0 features a unified, standardized, and commonly-used analysis workflow using Bismark (15) as the mapping algorithm. This standardized data processing facilitates data reusability and comparability. Furthermore, given the high usage of consensus reference methylomes from healthy people at different age groups provided in version 3.0, MethBank 4.0 increases the number of reference data from only one tissue (4577 samples) to 111 healthy tissues/cells (61 689 samples), which are normalized using the GMQN (16) to remove batch effects.

**Figure 1.**
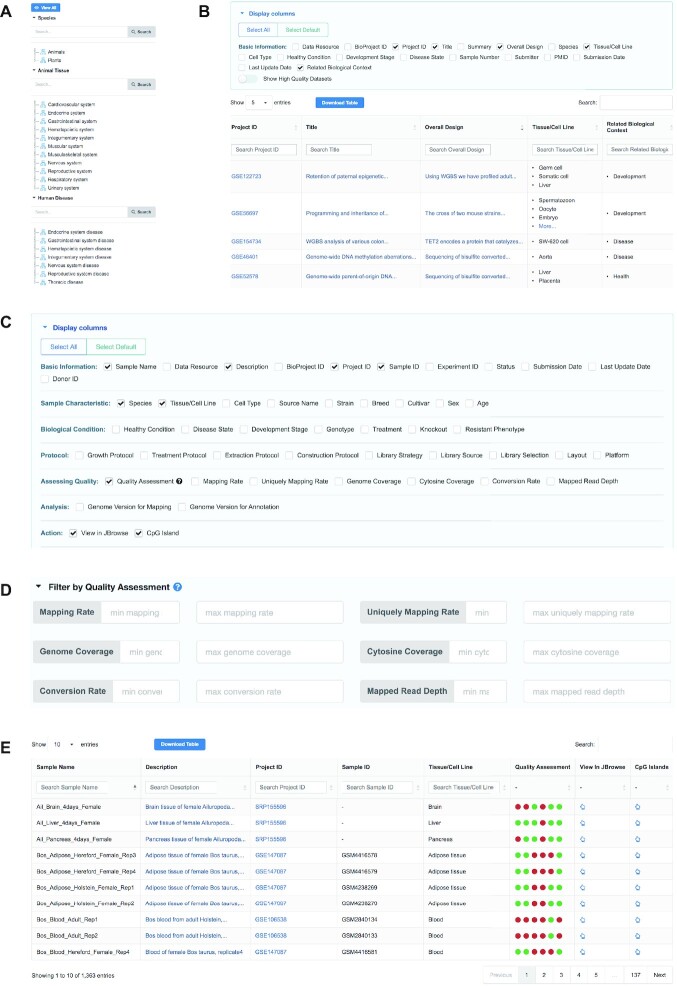
Screenshot of the page for projects and samples in MethBank 4.0. (**A**) Multiple tree structures in the browse page for datasets. (**B**) Manually curated meta-information in the page for projects. (**C**) Structural descriptive information in the page for samples. (**D**) Filter for samples quality by customized cut-off values in the browse page. (**E**) Intuitive tabular representation of description information for each sample.

In addition to the huge amount of data integration, MethBank 4.0 features a new structured curation model that integrates descriptive information about projects and adds detailed meta-information about samples in order to facilitate data presentation, exploration and visualization. Specifically, for each project, MethBank 4.0 summarizes a total of 18 metadata items with controlled vocabularies if appliable, including species, overall design, tissue/cell line, sample number, health condition, development stage, and disease state (https://ngdc.cncb.ac.cn/methbank/projects, Figure 1B). In an attempt to standardize the description of tissues/cell lines and diseases, manual curation was conducted for all projects by linking to controlled terms from Disease Ontology (17) and BRENDA Tissue Ontology (18). To help users retrieve and download the metadata of interest, projects were annotated and categorized into 15 biological contexts, which were standardized by biocurators using terms from controlled vocabularies. Based on these curated metadata for projects, MethBank 4.0 provides multiple tree structures to display all species, animal tissues, and human diseases (Figure 1A). All items in these structures are linked to the corresponding projects, assisting users in conveniently filtering projects of interest. Meanwhile, for users to access, understand, and use data more easily, MethBank 4.0 provides 45 structural items to standardize descriptive information about samples, including basic information, sample characteristic, biological condition, protocol, assessing quality, and analysis procedure (https://ngdc.cncb.ac.cn/methbank/samples, Figure 1C). To facilitate users to gain more intuitive insights into all collected samples, MethBank 4.0 not only provides structured metadata in a tabular form, but also categorizes experimental metadata under the framework of quality assessments, allowing users to filter and query samples according to their needs. Specifically, MethBank 4.0 evaluates the quality of all WGBS data based on 6 criteria, including mapping rate, uniquely mapping rate, cytosine coverage, genome coverage, mapped read depth, and bisulfite conversion rate, thus helping users to search for samples that meet the criteria through customized cut-off values (Figure 1D). To help users get a comprehensive and intuitive overview of the data, MethBank 4.0 provides a visualized representation of six quality criteria for each sample, with green representing compliance and red representing non-compliance. Meanwhile, users can interactively view the detailed quality information of the specific sample (Figure 1E).

**Figure 2.**
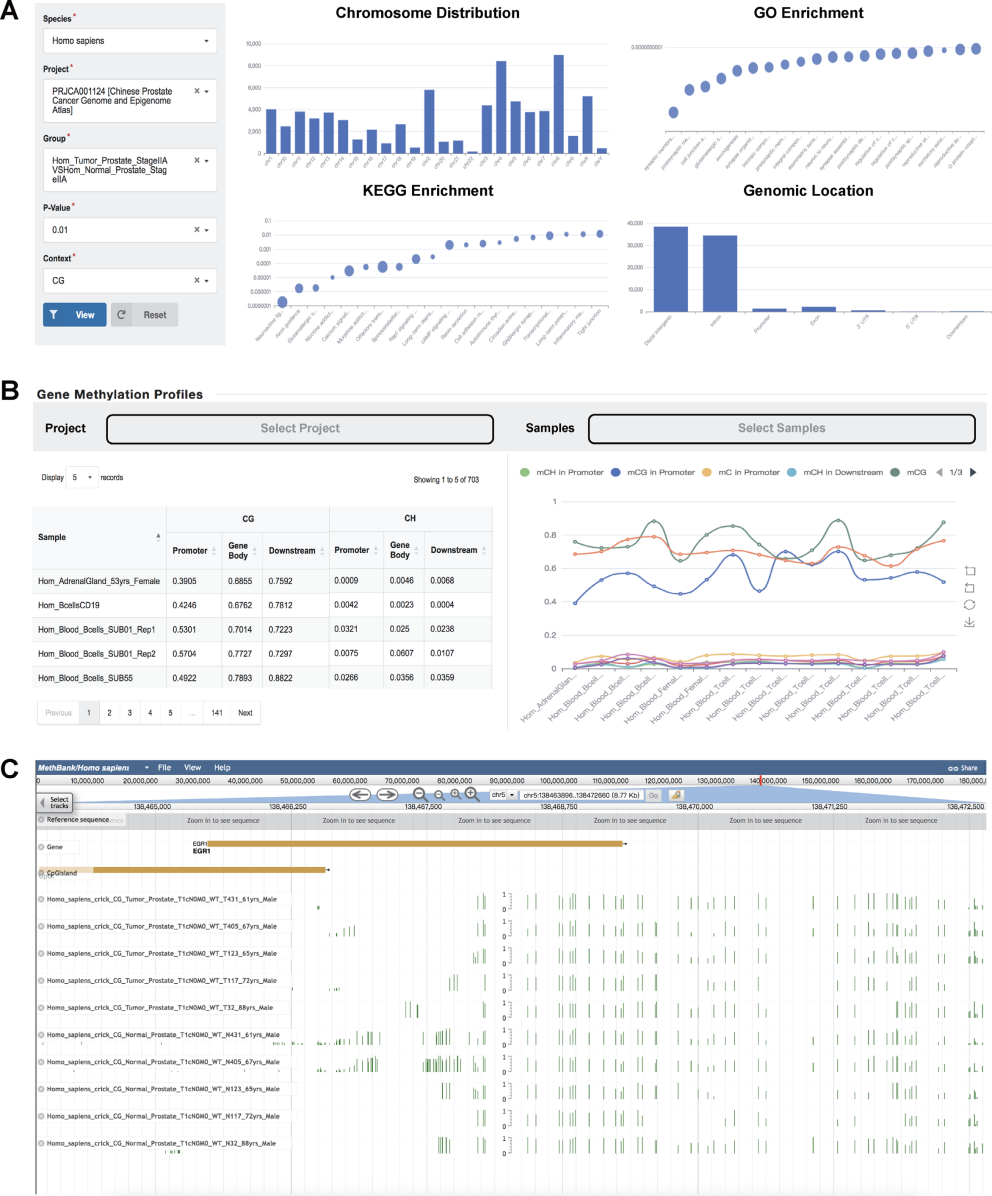
Annotations and visualizations of multiple methylation signatures of DMRs, genes, and mCpGIs. (**A**) DMRs Browser by specific project and biological context. Graphs display the functional annotation and pathway analysis of the DMRs. (**B**) Methylation profiles in promoter, gene body, and downstream of human gene ENSG00000169083 (*AR*) for different sequence contexts. (**C**) Visualization of methylation patterns of DMRs, genes, and mCpGIs in Genome Browser.

In order to help users to obtain a systematic, comprehensive, and intuitive landscape of DNA methylation signatures across multiple biological contexts, MethBank 4.0 extends differential DNA methylation analysis from differentially methylated promoters (DMPs) to DMRs and integrates value-added annotations derived from gene methylation profiles and mCpGIs. Studies have shown that genome-wide differential methylation patterns exist in various biological contexts, such as disease (19,20) and development (21,22), and this phenomenon is present in both animals (21,23) and plants (20,24). Therefore, DMRs between two or more biological contexts are considered as critical and underlying functional regions that may be involved in the transcriptional regulation of genes (25). As a result, MethBank 4.0 systematically identified 59 750 225 DMRs among 55 projects and 887 pairs of different biological contexts for 20 species. All DMRs were marked with features including genomic locations, associated genes, GO and KEGG enrichment (26,27) analysis results (Figure 2A). To further facilitate users to investigate the regulatory and functional roles of DMRs, MethBank 4.0 provides intuitive visualization of DMR downstream analysis that includes functional annotation and pathway analysis. Bar charts and bubble charts were used to visualize the enrichment of genomic regions and genes in the GO entries and KEGG pathways, respectively. Due to the relatively high proportion of methylated non-CG sites in plants (28), human embryonic stem cells (23), and brain (29), both CG and non-CG DMRs were processed by MethBank 4.0. By associating DNA methylation profiles of multiple biological contexts with specific gene functions, MethBank 4.0 aims to assist users in screening potential methylation biomarkers as well as exploring the regulatory mechanisms of DNA methylation.

Beyond that, MethBank 4.0 optimizes the function of gene methylation profiles, enabling users to explore the methylation status and variation of genes in specific biological contexts (Figure 2B). Specifically, MethBank 4.0 simultaneously displays gene methylation profiles in promoter, gene body, and downstream for different sequence contexts on a single image by selecting a project or multiple samples of interest, which will help users conveniently investigate gene-specific methylation status across diverse biological contexts. Additionally, the previous version of MethBank detected genes related to mCpGIs for all samples, but taking account of site-specific hypermethylation, especially at promoter CpGIs, may suppress the expression of the downstream genes (30,31), MethBank 4.0 additionally annotates mCpGIs in genome elements and visualizes downstream analysis results. All images can be downloaded in standard formats and used directly for publication. Notably, MethBank 4.0 is equipped with an interactive genome browser with custom tracks, thus allowing users to visualize the methylation patterns of genes, mCpGIs, and DMRs for existing data and facilitating straightforward side-by-side comparisons across samples (Figure 2C). DMRs, together with brand-new gene methylation profiles and mCpGIs, constitute an essential resource to help users systematically investigate DNA methylation signatures in epigenetic studies.

### Knowledge modules

#### Featured DMGs

Up until now, the pilot experiments of genome-wide DNA methylation research have described several characteristic methylation features in various biological contexts. These features, especially DMGs, show great value for serving as biomarkers and revealing functional differences, but are difficult to extract and summarize because they are scattered over thousands of publications.

**Figure 3.**
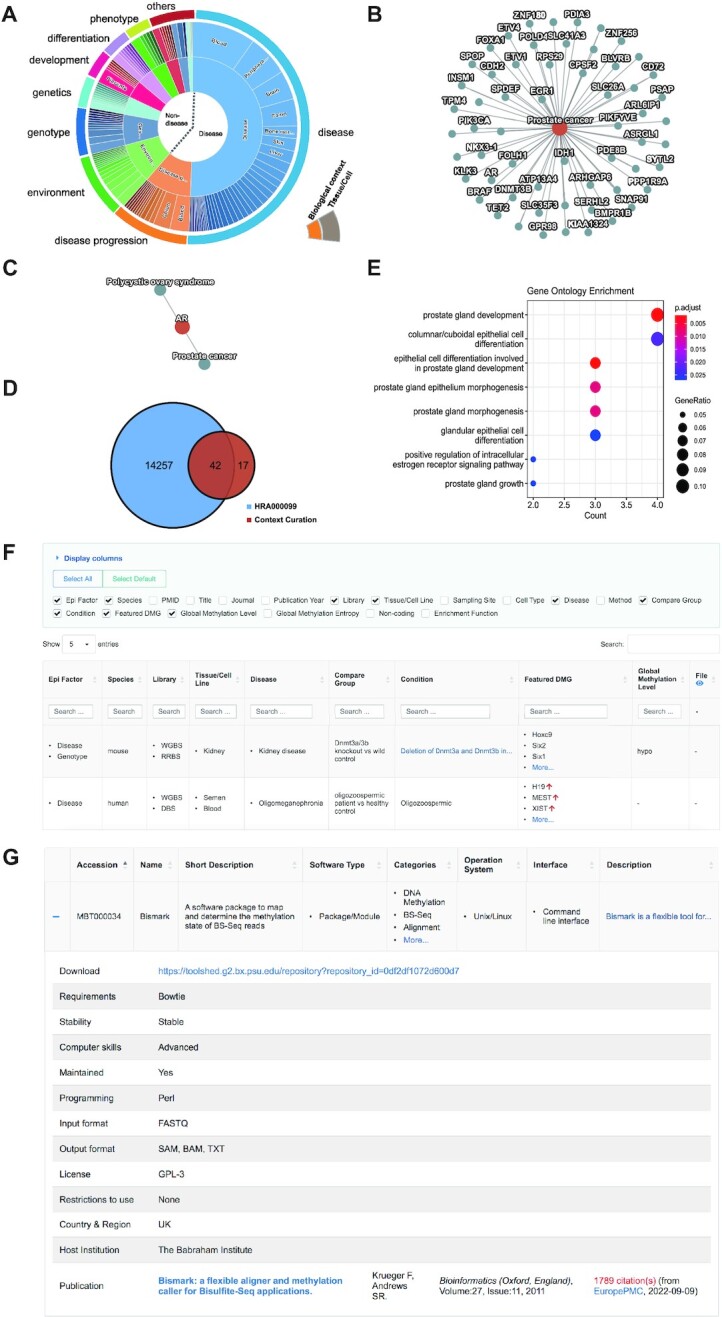
The Knowledge modules of MethBank 4.0. (**A**) Proportions of context curation publications as defined in the two levels accounting figure, covering 11 biological contexts and 77 tissues/cell lines. (**B**) Network diagram of diseases, where the red nodes represent diseases and the grey nodes represent relevant DMGs in the Featured DMGs module. Fifty-nine genes associated with prostate cancer are shown in the example. (**C**) Network diagram of genes, the example shows two diseases associated with gene *AR*, prostate cancer and polycystic syndrome. (**D**) Forty-two of the 59 manually curated genes associated with prostate cancer are overlapped with DMGs obtained from the analysis in the Data module. These 42 genes were significantly enriched in prostate cancer-related pathways as shown in Figure (**E**). (**F**) Screenshot of the web page in the Featured DMGs module. (**G**) Screenshot of the web page in the Tool Collection module, taking Bismark as an example.

In MethBank 4.0, the Featured DMGs module summarizes featured DMGs associated with biological contexts via full-scale manual curation to increase the potential availability of retrieving epigenetic marker genes and shared properties for various biological contexts. The full text of all publications in the past twelve years (2010-present) with the keyword matching ‘WGBS’, ‘whole-genome bisulfite sequencing’, ‘RRBS’, and ‘whole-genome DNA methylation’ were initially retrieved from PubMed (https://pubmed.ncbi.nlm.nih.gov/) for human and mouse. We manually reviewed 1039 publications, of which 228 were retained in relation to the featured DMGs. These uniformly curated data are mainly enriched with DMGs correlated to 11 biological contexts including disease, disease progression, development, differentiation, phenotype comparison, genotype, parental genetics, aging, treatment, environmental factor, and tissue comparison (https://ngdc.cncb.ac.cn/methbank/curation, Figure 3A). The Featured DMGs module focuses on genes tend to be the top significantly different subsets, potential markers, hub genes among networks, driver genes in diseases, and experimentally validated genes related to expression levels. Notably, MethBank 4.0 constructs a network query of 128 diseases and corresponding featured genes to enhance gene-disease knowledge discovery of biomarkers (Figures 3B and C). Taking prostate cancer as an example, there are 59 featured DMGs curated from 5 publications (Figure 3B) (32–36). Among them, there are 42 intersections with differential analysis results of project HRA000099 in the Data module of MethBank 4.0, primarily including the key androgen-responsive genes such as *EGR1*, *AR*, *KLK3*, and *FOLH1* (32,33,36) (Figure 3D). These curated DMGs are enriched in prostate cancer-related pathways including prostate gland development and columnar/cuboidal epithelial cell differentiation (Figure 3E). Meanwhile, the search for genes provided epigenetic signatures shared between various disease types, such as androgen receptor *AR* in the network associated with two disease nodes (Figure 3C), viz., prostate cancer and female polycystic ovary syndrome, agreeing well with previous findings that the balance of androgen activity is closely linked with human reproduction (37,38).

Besides those featured DMGs, the DMRs/DMGs list files are also re-organized in MethBank 4.0. The relevant genomic distribution and detailed changes of DNA methylation levels for those context-associated DMGs are accessible in tabular format (Figure 3F). Altogether, the Featured DMGs module collects DMGs from numerous studies and biological contexts, providing valuable resources in aid of in-depth investigations of epigenetic regulation mechanisms.

#### Tool collection

More and more tools are being developed for methylation data analysis as the field of methylation research has evolved rapidly. Therefore, it is particularly important to help researchers choose the applicable ones from a vast array of analysis tools. To tackle this issue, the Tool Collection module was added in MethBank 4.0 to organize these accessible tools for quick and accurate selection. Users can quickly filter and find suitable tools for their research needs by searching for keywords. As of June 31, 2022, the Tool Collection module collects 501 methylation tools summarized using 25 normalized metrics including description, category, sequencing platform, programming language, configuration requirement, documentation, access method, publication, citation, and others (https://ngdc.cncb.ac.cn/methbank/methtool, Figure 3G). These tools were categorized based on the analysis tasks, such as differential methylation analysis, normalization, visualization, and batch effect correction. In addition, the Tool Collection module tracks citations of tools regularly. Overall, the Tool Collection module provides a valuable resource for researchers to quickly and accurately locate the methylation analysis tools they need.

### Tools module

In the previous version, both Age Predictor for estimating the age of DNA methylation and IDMP for identifying DMPs were developed. Considering that the identification of DMRs between paired samples is one of the most important analysis of WGBS data (39), MethBank 4.0 additionally provides a one-stop analysis tool named DMR Toolkit (https://ngdc.cncb.ac.cn/methbank/tools/dmr/toolkit) to identify, analyze, and visualize DMRs of 20 species. DMR Toolkit can help users to perform differential methylation analysis in the most convenient way. With only one simple required input operation, DMR Toolkit can conduct the identification of DMRs with genomic element annotation and related gene function enrichment, as well as the corresponding visualization charts. Notably, DMR Toolkit provides both CG and non-CG DMRs at different *P*-values (0.01 and 0.05) using DSS (40), which will help users characterize epigenetic signatures and potential biomarkers on multiple species.

## CONCLUSION AND PERSPECTIVES

As one of the core resources of National Genomics Data Center, China National Center for Bioinformation (41), MethBank is a database of high-quality whole-genome single-base resolution DNA methylation data. Over the past few years, MethBank has been continuously evolving to keep up with the ever-growing data in this community and has been considered as an important repository for epigenetic studies. The new version 4.0 shows the rapid increase in data volume relative to any previous version, greatly expandings the range of species and biological contexts as well. Compared to the previous version, MethBank 4.0 emphasizes downstream reanalysis of WGBS data and considerably enriches the differential methylation analysis between biological contexts. Another highlight of MethBank 4.0 is the newly added Knowledge modules that contain manually curated information of featured DMGs associated with biological contexts and methylation analysis tools. The new version additionally includes a one-stop analysis tool for differential methylation analysis across multiple species. To meet the needs of growing data and visualization of downstream analysis, the new version also reorganizes the data structure and web interface, resulting in a more comprehensive resource to enhance data utilization.

In addition to incorporating more data and equipping more enhanced functionalities, we plan to develop tools to eliminate the batch effect, which will make it possible to integrate more data from various sources. Meanwhile, we will link closely with our self-developed databases EWAS Open Platform (42) for array-based DNA methylation data and scMethBank (43) for single-cell sequencing-based DNA methylation data, with the aim to help users systematically decipher the regulatory mechanisms of DNA methylation from multiple resolutions and dimensions.

## DATA AVAILABILITY

MethBank is a database of DNA methylomes across diverse species. All data, software and resources are available at https://ngdc.cncb.ac.cn/methbank/. Any queries, comments, and suggestions on MethBank can be provided by email via methbank@big.ac.cn.
